# Targeted cognitive game training enhances cognitive performance in multiple sclerosis patients treated with interferon beta 1-a

**DOI:** 10.1186/s12984-021-00968-3

**Published:** 2021-12-19

**Authors:** Shay Menascu, Roy Aloni, Mark Dolev, David Magalashvili, Keren Gutman, Sapir Dreyer-Alster, Franck Tarpin-Bernard, Ran Achiron, Gil Harari, Anat Achiron

**Affiliations:** 1 Multiple Sclerosis Center, Sheba Medical Center, Tel-Hashomer, Ramat Gan, Israel; 2 Sackler School of Medicine, Tel-Aviv University, Tel Aviv-Yafo, Israel; 3 Department of Behavioral Sciences and Psychology, Ariel University, Ariel, Israel; 4 HAPPYneuron, Villeurbanne, France; 5 School of Public Health, University of Haifa, Haifa, Israel; 6 Laura Schwarz-Kipp Research of Autoimmune Diseases, Sackler School of Medicine, Tel-Aviv University, Tel Aviv-Yafo, Israel

**Keywords:** Multiple sclerosis, Cognition, Information processing speed, Executive function, Games, Training, Interferon-beta-1-a, Plasticity

## Abstract

**Background:**

Prevention of cognitive decline in Multiple Sclerosis (MS) is of major importance. We explored the effect of a 6 months computerized game training program on cognitive performance in MS patients with mild cognitive impairment.

**Methods:**

This was a single-center, randomized prospective study. We enrolled in this study 100 eligible MS patients treated with Interferon-beta-1a (Rebif). All had mild cognitive impairment in either executive function or information processing speed. Patients were randomized 1:1 to either use the cognitive games platform by HappyNeuron (HN) or receive no intervention. Executive function and information processing speed scores were measured at 3 and 6 months from baseline to evaluate the effect of game training on cognitive scores.

**Results:**

In both executive function and information processing speed, the game Training group showed significant improvement after 3 and 6 months. The Non-Training group showed mild deterioration in both domains at 3 months, and further deterioration that became significant at 6 months in executive function. Furthermore, at 6 months, the percent of patients in the Training group that improved or remained stable in both cognitive domains was significantly higher compared to the Non-Training group.

**Conclusions:**

Our findings suggest that cognitive game training has a beneficial effect on cognitive performance in MS patients suffering from mild cognitive impairment. While further evaluation is required to assess the longevity of that effect, we nonetheless recommend to MS patients to be engaged in cognitive gaming practice as part of a holistic approach to treating their condition.

**Supplementary Information:**

The online version contains supplementary material available at 10.1186/s12984-021-00968-3.

## Introduction

Cognitive impairment is reported to occur in multiple sclerosis (MS) patients along the disease course [1] and can present even in the early disease phase [2, 3].

However, only a few studies assessed the effect of cognitive rehabilitative practice by cognitive training on cognitive performance in MS using various models of cognitive games to improve brain plasticity [4–7]. A recent systematic review and meta-analysis [8] assessing the effects of computerized cognitive training on cognitive outcomes in adults with MS found small to moderate effect sizes for attention/processing speed, executive functions, and verbal and visuospatial memory. These key cognitive domains are the ones most commonly affected in MS-related cognitive impairment [9, 10].

The scientific support for the beneficial outcome of brain training, defined as a process that enhances a cognitive skill through engagement in a particular cognitive exercise [11], thrives on the expected effects of neuroplasticity. Neuroplasticity encodes the ability of the nervous system to respond to intrinsic and extrinsic stimuli by reorganizing its structure, function, and connections. Such plasticity is adaptive when associated with a gain in function [12]. Moreover, repetitive training is reported to trigger neural changes within a short period [13].

The value of enriched environments in enhancing brain recovery at both structural and chemical levels has been repeatedly demonstrated in animal studies [14, 15]. These studies illustrated that environments facilitate enhanced cognitive, motor, and sensory stimulations mainly by applying animal housing conditions with complex arrangements of toys, platforms, and tunnels being changed every few days, shown to promote motor learning and memory. In accordance, in humans recovering from brain injuries, enriched environments promote better motor recovery and augment neuronal plasticity within non-injured, functionally connected brain regions [16, 17]. The rationale for motor and sensory training is based not only on direct activation of the weakened muscles or awakening sensory stimuli in a numb limb but also on the possibility that training will enhance neuroplasticity by affecting cellular mechanisms that determine myelin sheath extension and stabilization around axons. It is conceivable to hypothesize that neuroplasticity may be enhanced by cognitive training. Cognitive game training is an innovative approach that aims to improve cognitive skills by repeated sets of targeted gaming exercises. Using cognitive gamification might help overcome one of the core challenges of conventional cognitive rehabilitation, that of motivation to train; these cognitive tasks tend to be highly repetitive with a need for multiple training sessions and often bore the patients and reduce compliance. The ability to train cognitive aspects using computerized gamification approaches that transform conventional tasks into a more entertaining though no less difficult cognitive practice, can maximize user engagement and result in better cognitive outcomes, specifically in patients at high risk for cognitive decline [18, 19]. Moreover, web-based cognitive games are easily accessible and have already demonstrated a benefit among healthy [20], older adults [21], and adults with chronic acquired brain injury [22]. We have recently reported the correlations between web-based cognitive game training tasks and cognitive performance in patients with multiple sclerosis and demonstrated the correlations between specific games and cognitive performance [23]. In the current prospective study, we further evaluated a large group of MS patients with mild cognitive impairment, all treated with the same immunomodulatory drug, and assessed whether targeted cognitive game training for a 6 months period provides cognitive benefits within the domains of information processing speed and executive functioning.

## Methods

### Study design and patient population

This was a single-center, randomized prospective study, conducted in the Multiple Sclerosis Center, Sheba Medical Center, Tel Hashomer, Israel. Ethical approval to conduct the study was granted by the Sheba Medical Center Ethics Committee (Institutional Review Board number SMC-333016), and each participant signed written informed consent. Data were collected between May 2016 and February 2021. Data is available upon request from the authors of this study.

Inclusion criteria were as follows: (1) Patients diagnosed with relapsing–remitting multiple sclerosis or secondary-progressive multiple sclerosis according to revised McDonald Criteria 2010 [24]. (2) Age between 18 and 55 years. (3) Cognitive impairment, defined as a cognitive test performance in information processing speed or executive function of a score < 95. This cut off was chosen to exclude patients with high cognitive performance, while including patients with relatively mild impairment, who could most likely benefit from cognitive training. (4) Current treatment with Rebif® and RebiSmart 2.0 (in doses of 22 or 44 mcg subcutaneous, three times a week) (5) Signed written informed consent.

Exclusion criteria were as follows: (1) Steroid treatment within the last 3 months. (2) Severe cognitive decline that prevents cognitive assessment. (3) Significant depression and/or anxiety. (4) Visual impairment or upper extremity motor disability the precludes the ability to perform cognitive training. Demographic and clinical data were collected for all of the study subjects and included age, gender, years of education, disease duration, and disability status as measured by the Expanded Disability Status Scale (EDSS) [25].

### Cognitive assessments

Cognitive assessment was performed using the computerized MindStreams Global Assessment Battery (NeuroTrax Corp., Bellaire, TX, USA), which has been validated in MS population [2], and was reported to have good reliability and validity relative to paper-based tests [9]. Executive function and information processing speed domains were evaluated, as they reflect the most vulnerable cognitive impairments in MS patients. Outcome parameters accuracy and response time were normalized for age and education according to stratifications of a normative database of cognitively healthy subjects to fit an IQ-like scale (mean: 100, SD: 15). Value of 95 was defined as the cutoff for mild cognitive impairment in either executive function or information processing speed domains.

### Randomization

Patients eligible for the study signed written informed consent and were randomized to either the game training or the non-game training group. Randomization was performed for cognitive performance and EDSS which were the major important variables to ensure similar cognitive performance and disease characteristics before cognitive game practice. We did not include a sham group in the study, due to the difficulty of constructing a sham practice that does not stimulate any cognitive function.

### Sample size calculation

A sample size calculation demonstrated that a sample of 42 subjects in each group will yield 80% power to detect a difference in means of 5.000 (the difference between a Group 1 mean, m1, of 0.0 and a Group 2 mean, m2, of −5.0) assuming that the common standard deviation is 8 using a two-group t-test with a 0.050 two-sided significance level.

The expected drop-out rate in this study was 20%, hence, we aimed to enroll 48 subjects in each group.

### Cognitive neuro-game training

Cognitive neuro-game training using HappyNeuron platform (HN, https://www.happyneuronpro.com/en) was performed by the patients randomized into the training group twice weekly, each session lasting 30 min, for 6 months (a month calculated as 28 days). HappyNeuron platform is a web-based cognitive training game platform. Nine cognitive exercises developed by Happyneuron for cognitive training were applied for practice during this study. The HAPPYneuron brain training cognitive game-platform is aimed to stimulate various cognitive aspects including memory, attention, language, executive functions (reasoning, logical thinking), and visual and spatial skills. Specifically, we included nine cognitive games related to executive functions, e.g., Turn around, Points of View, Basketball in New York, Sleight of Hands, Towers of Hanoi, and information processing speed, e.g., Sleights of hands, Entangled Figures, Towers of Hanoi, Under Pressure, Shapes and Colors, Private Eye Turn around, Points of View. A detailed description of each game can be found in Additional file 1: Table S1. For the purpose of the current study, we have defined the same level of difficulty for each game to all participants. Each patient was asked to complete all nine cognitive games in the same order using the computer keyboard. For each cognitive game, average accuracy and response time were recorded.

### Game training adherence assessment

The adherence rate in the game training group was assessed weekly using the HN platform to ensure patients keep training twice weekly for 30 min’ sessions. Subjects that missed more than 20% of the training sessions over time were excluded from the analysis.

### Statistical analysis

All measured variables and derived parameters were tabulated by descriptive statistics. Paired T-test (paired observations) was applied for testing the statistical significance of the change in the executive function and in information processing speed from baseline at month 3 and month 6 within each study group.

The two-sample T-test was applied to compare the relative change in executive function and information processing speed from baseline between Training and Non-Training groups at month 3 and month 6.

A linear model for repeated measures was applied for analyzing the difference between Training and Non-Training groups in executive function change and in information processing speed change from baseline at any time, including the fixed effect time and adjusted for baseline.

Chi-Square Test was applied to compare the frequency of improvement in executive function and in information processing speed between Training and Non-Training groups at month 3 and month 6.

The data were analyzed using the SAS ® version 9.4 (SAS Institute, Cary North Carolina).

## Results

### Demographics

164 subjects were evaluated for eligibility, 100 of which fulfilled all inclusion criteria and were included in the study. 51 subjects were randomized into the Training group, and 49 into the Non-Training group. Ninety-three subjects completed the 3-month follow up, and 87 subjects completed the 6-months follow up.

Clinical and cognitive variables of the study participants by group are presented in Table 1.

**Table 1:** Clinical and cognitive variables of the study participants by group

Mean ± SD Median	All N = 100	Training N = 51	Non-training N = 49	p
Age, years	43.8 ± 7.29 45.7	41.8 ± 7.9 42.6	46.0 ± 6.0 47.1	< 0.001
gender, F:M	74:26	36:15	38:11	
Education, years	14.2 ± 2.31 14	14.5 ± 2.31 14	14.0 ± 2.30 14	0.136
Disease Duration, years	14.2 ± 7.93 13.6	12.7 ± 7.92 11.4	15.7 ± 7.71 16.0	< 0.001
EDSS	2.6 ± 1.70 2.0	2.7 ± 1.69 2.0	2.65 ± 7.93 2.0	0.346
Interferon-beta-1A Tx duration, years	6.7 ± 4.48 5.5	5.3 ± 3.58 4.2	8.2 ± 4.87 7.6	< 0.001
Executive function	91.8 ± 12.74 93.5	91.4 ± 12.59 93.0	92.2 ± 13.02 95.0	0.367
Information processing speed	84.7 ± 14.07 85.0	86.0 ± 13.0 88.0	83.3 ± 15.14 82.0	0.174

Randomization was performed only for cognitive performance and EDSS which were the major important variables to ensure similar cognitive performance and disease characteristics before cognitive game practice.

At baseline, performance in executive function and information processing speed did not differ between the Training and Non-Training groups, scores being 91.4 vs. 92.2 and 86 vs. 83.3, respectively.

### Effect of targeted cognitive game training on cognitive performance over time

**Fig. 1:**
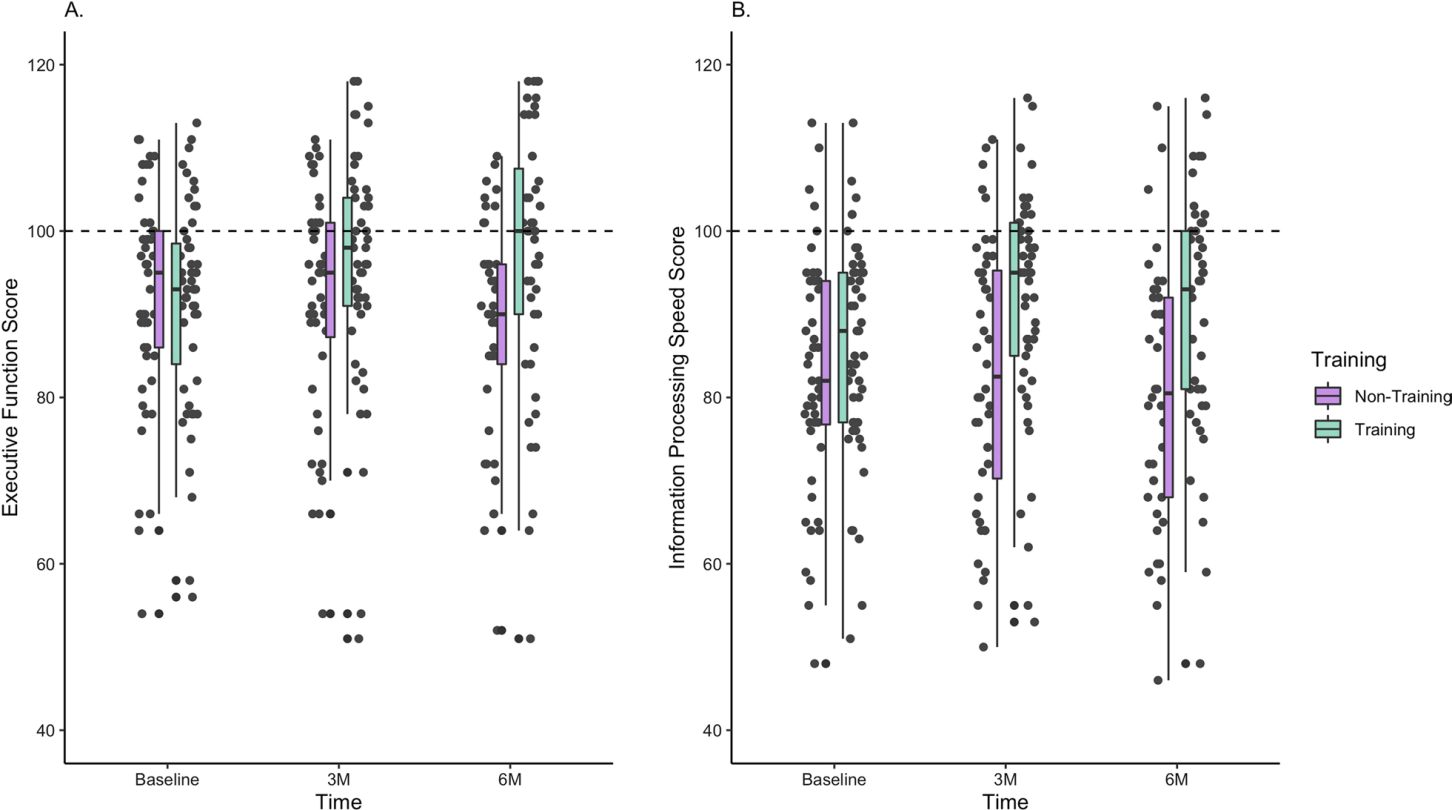
Scatter and box plots at baseline, 3 and 6 months, **A** Executive function scores in Training (light green) and Non-Training (purple) groups. **B** Information processing speed in Training (red) and Non-Training (blue) groups. Box plots include median, IQR, minimum and maximum values

The results of targeted cognitive game training on executive function and information processing speed over time are shown in Fig. 1A and B, respectively.

### Executive function

At 3 months from baseline, mean (± SD) score in executive function was 95.9 ± 14.0, and 91.8 ± 13.7 for the Training and Non-Training groups respectively (p = 0.157 for unpaired T-test). At 6 months, executive function scores were 97.6 ± 16.0, and 88.3 ± 13.5 for the Training and Non-Training groups respectively (p = 0.005). Linear model for repeated measures adjusted to baseline demonstrated that the change in executive function over time was significant between groups, p = 0.026.

The Training group demonstrated a statistically significant improvement in executive function after 3 and after 6 months (paired T-test, p = 0.0035 and p = 0.0007, respectively). The Non-Training group showed mild but non-significant deterioration in executive function at 3 months (p = 0.1634), and further decline that became statistically significant at 6 months (p = 0.0036).

### Information processing speed

At 3 months from baseline, mean (± SD) score in information processing speed was 91.3 ± 14.0, and 82.7 ± 16.2 for the Training and Non-Training groups respectively (p = 0.007 for unpaired T-test). At 6 months, information processing speed scores were 92.5 ± 16.6, and 80.2 ± 15.8 for the Training and Non-Training groups respectively (p < 0.001).

Linear model for repeated measures adjusted to baseline demonstrated that the change in information processing speed over time was significant between groups, p = 0.017.

The game training group demonstrated a statistically significant improvement after 3 and after 6 months (paired T-test, p = 0.0013 and p > 0.001, respectively). The non-training group showed mild deterioration in information processing speed performance that was not significant at 3 months (p = 0.4978) and further mild decline at 6 months (p = 0.0687).

### Effect of targeted cognitive game training on the frequency of patients with stable or improved cognitive performance over time

**Fig. 2:**
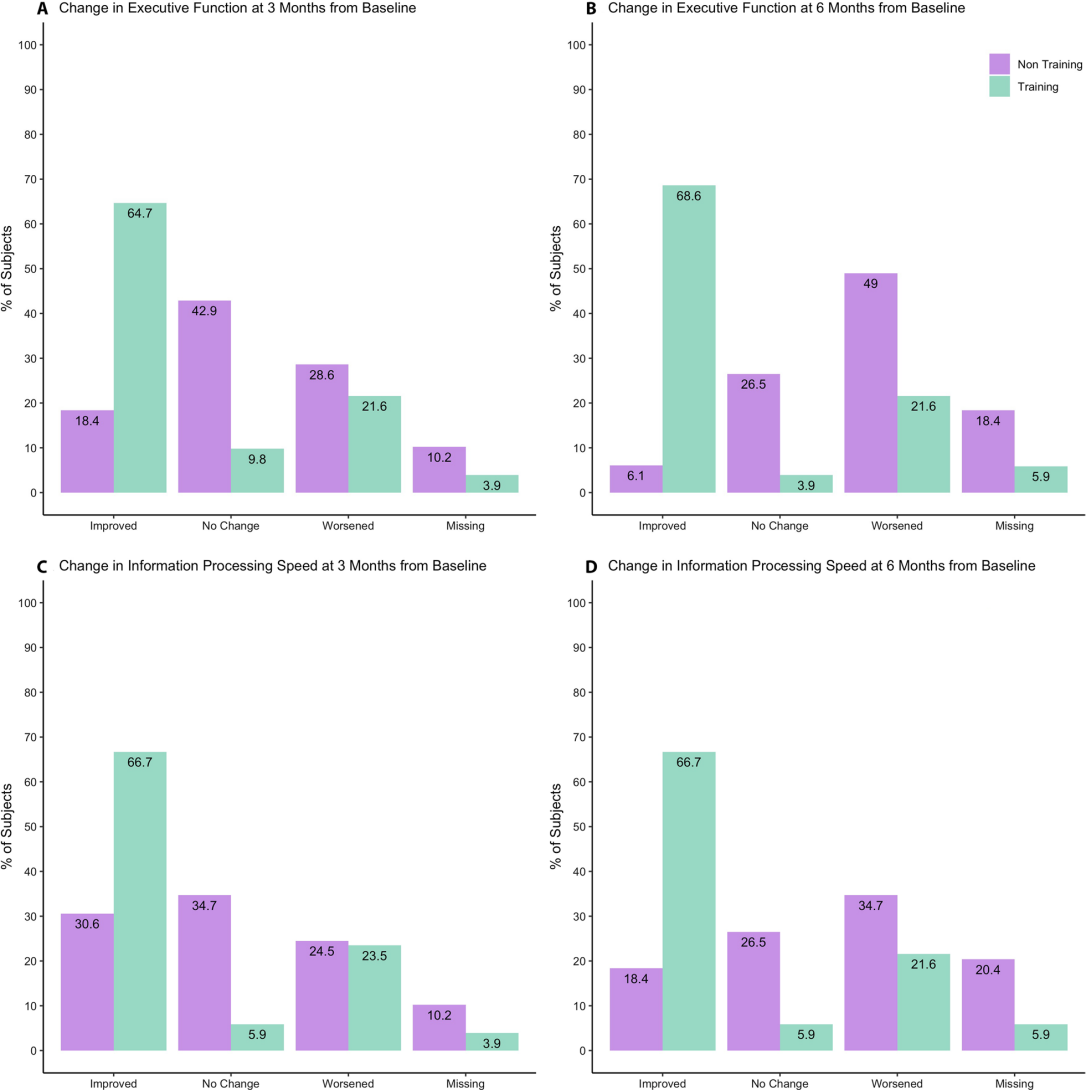
Frequency of patients who improved/remained stable, or worsened in executive function at 3 months (**A**) and 6 months (**B**) from baseline, and information processing speed score at 3 months (**C**) and 6 months (**D**) from baseline, in Training and Non-Training groups

The frequency of patients that either improved/remained stable, or worsened in cognitive performance over time in relation to targeted cognitive training are shown in Fig. 2A–D.

For both cognitive domains, no statistically significant differences were observed regarding the frequency of improvement in patients at 3 months from baseline (p = 0.28 and p = 0.45 for executive function and information processing speed, respectively). However, at 6 months, the percent of patients in the Training group that improved or remained stable in both cognitive domains was significantly higher compared to the Non-Training group (p < 0.001 and p = 0.01 for executive function and information processing speed, respectively).

### Adherence to the cognitive game training

The majority of MS patients in the Training group had a good compliance rate; 13.7% of the patients missed 5 sessions during the 3 months training, and 19.6% missed 5 sessions during the 3 to 6 months training sessions.

## Discussion

Targeted cognitive training holds promise in promoting cognitive performance in patients suffering from MS, and specifically in young MS patients with mild cognitive decline. These subjects have the ability to use computerized gaming and can easily practice; moreover, the variety and diversity of the various games, along with the option to gradually increase the difficulty of the games to match each participant’s abilities, constitute major advantages for the rehabilitation process that enable personalized targeted cognitive training, an aspect shown to increase its effectiveness [26]. Developing targeted tools such as cognitive game training platforms to detain cognitive decline is of significant importance for these patients, as they may develop cognitive decline at an early disease stage. One of the pressing questions is how to assess the effectiveness of various cognitive game training systems in real-world settings and the possible improvement in cognitive performance while considering age and educational level of users, system and environmental constraints, type of feedback, and adherence to training. In the current study, we employed an interactive game training exercise system that was targeted to patients with MS suffering from mild cognitive impairment in either executive function or information processing speed and assessed their cognitive performance after 3 and 6 months of practice, in comparison to disability- and cognitive-performance matched group of MS patients that did not practice the targeted cognitive game training platform. In our previous study, we demonstrated that cognitive decline first occurs at 5 years from onset, suggesting the existence of a therapeutic window during which patients may benefit from interventions to maintain cognitive health [2]. In the current study, all patients had a longer disease duration (median 14.2 years), beyond the cognitive protective 5-year window.

Our findings demonstrated that MS patients that practiced cognitive training improved their cognitive performance within a period of six months of practice. Executive function performance significantly increased at as early as 3 months, while in non-training patients, executive function performance significantly decreased at 6 months. Information processing speed significantly increased over 3 and 6 months, while in non-training patients, information processing speed performance mildly decreased at 6 months. These findings imply that game training can contribute to better performance in daily activities that require these cognitive capabilities.

The compliance rate for training was relatively high, suggesting that the gamification approach is effective in improving patients’ motivation and enjoyment while practicing. The ability to remind patients to participate in the training and assess their compliance was unique to the current study. This was achieved through the HN computerized platform and the ability to evaluate the training sessions by remote control.

Previous studies in older populations with impaired cognition showed that cognitive training can produce moderate to large beneficial effects on memory related-outcomes [27], and enhance cognitive control [21], due to brain plasticity-related mechanisms [28, 29]. The only study that assessed the effect of a 3-month cognitive training program in MS patients with mild-to-moderate cognitive impairment, included 30 subjects that received a computer-assisted neuropsychological training program and 32 subjects that were randomized to no intervention [30]. The rehabilitation program focused on attention, processing speed, memory, and executive function through computerized and paper and pencil tasks. Similar to our findings, the computer-assisted neuropsychological training group showed significant improvements in memory and phonetic fluency while the non-training group did not show similar changes. In their study, no data were provided concerning immunomodulatory treatment and adherence to the cognitive training.

The limitation of our study is that we did not evaluate the longevity of the cognitive training effect after cessation of training. We suspect that without practice the effect will not be sustained, similarly to any physical exercise or training activities whereby practice is mandatory. Future studies are recommended to evaluate the longevity of the effect achieved by cognitive training once the patients cease practicing. Understanding the scope of training needed to sustain the effect will assist in better composing evidence-based recommendations to patients regarding cognitive training schedule.

A further limitation is the difference in baseline parameters of age, disease duration, and treatment duration in the study population, as randomization was performed to adjust for cognitive performance and neurological disability. Generally, the training group consisted of younger patients, with shorter disease and treatment duration. The effect of these differences on the results is difficult to assess, and future studies should consider adjusting for these characteristics as well.

To the best of our knowledge, this is the first study to directly compare the correlations between cognitive training games and cognitive assessment in a large cohort of patients for 6 months. The finding that targeted cognitive game training significantly improved performance of both information processing speed and executive function is encouraging, suggesting that game tasks involving both planning and decision-making elements can contribute to cognitive performance in day-to-day activities. Our findings may indicate the importance of targeted cognitive rehabilitation for young patients with brain damage leading to mild cognitive impairment. Our data demonstrates that six months of cognitive training conducted twice a week will result in a beneficial effect and preserve performance for a period of 6 months, we therefore recommend to MS patients to be engaged in cognitive gaming practice as part of a holistic healthcare approach that integrates many cognitive aspects in daily life, e.g. chess playing, solving puzzles or sudoku, and computerized gaming, to prevent cognitive deterioration and enhance neural plasticity.

## Conclusions

In conclusion, in this prospective study we showed that targeted cognitive game training significantly improved performance of both information processing speed and executive function in MS patients within 6 months of training. These findings suggests a beneficial effect of game training in the rehabilitative process of patients suffering from MS.

## Supplementary Information

**Additional file 1: Table S1. **Description of cognitive training games and level of performance.

## Data Availability

The datasets used and/or analyzed during the current study are available from the corresponding author on reasonable request.

## Abbreviations

MS: Multiple Sclerosis
HN: HappyNeuron
EDSS: Expanded Disability Status Score
