# Supplementary material for: Targeted cognitive game training enhances cognitive performance in multiple sclerosis patients treated with interferon beta 1-a
Source: J Neuroeng Rehabil. 2021 Dec 19;18:175. doi: 10.1186/s12984-021-00968-3 (PMC8684659; doi:10.1186/s12984-021-00968-3)
Supplement: Supplementary file 1 — Additional file 1: Table S1. Description of cognitive training games and level of performance. [file 12984_2021_968_MOESM1_ESM.docx]

**Table S1:** Description of cognitive training games and level of performance

| Game | Description of task | Designed level |
| --- | --- | --- |
| Turn around | Determine if the 3-D geometrical figures  presented from various angels of view are the same or are different geometrical figures. | Number of series – 4  Number of objects - 2  3-D object presentation – simultaneous Complexity of the object – medium Memorization time - unlimited  Answer time – 20 msec  No reminder of objects. |
| Points of View | The task is divided into two parts. In the first part, the patient has to deduce the location of an  observer on a map based on what he sees. In the  second part, the patient has to deduce what the observer sees based in his location on a map. | Number of series – 6  Type of game - What do you see  Number of figures – 5  Color of figures – different  Number of points of view – 4  Number of tries – 2  Response time – 30 |
| Basketball in  New York | The patient will see a first line of three hoops with colored basketballs inside. In the second line, he will have to mentally determine the number of basketball-moves required to reach the same configuration as the first line of hoops. | Number of configurations- 5  Number of balls – 4  Levels of difficulty – medium  Response time – 40  Five different colored basketballs are shown. |
| Sleight of Hands | The patient needs to decide whether the hand which appears briefly on the screen is a left or a right hand. A mirror images are applied as well. | Number of hands – 20  Type of hands – alone/in action  Hand presentation - normal/ mirrored  Display time - 5 sec  Answer time - 5 sec |
| Towers of Hanoi | The patient must configure colored rings on a series of pages in order to match a target. The rules: Can move the top-most ring in each peg to another peg, but only one ring at a time and never put a larger ting on top of a smaller ring. | Number of moves - 5 to 9  Number of rings - 5  Number of tries - 3  Number of configuration - 3  Allowed time - 3 minutes. |
| Entangled Figures | The patient sees three entangled objects, which, taken together, make up a more complex figure. These three entangled graphical objects are compared to a multiple choice of similar or different objects that are possible components of the complex entangled figure. Patient must choose the ones that are part of the entangled figure. | Type of figure - figurative drawing  Number of figures - 3  Appearance of figures - staggered Theme of figures – unique Memorization time -10 sec  Time allowed to select the figures composing the  entangled figure - 20 sec  Five entangled figures are presented for each level. |
| Private Eye | The patient needs to find a symbol that was shown before starting the task, in a grid full of intricate symbols and letters (intruder) that differs from other symbols (all of which identical). | Type of help - multi- symbols  Size of the grid - 4x6  Number of sequenced series – 5  Types of character – symbols  Response time – 20 sec. |
| Shapes and Colors | Memorize several figures of various shapes and colors and then recognize them among slightly different ones. | Type of figures - shapes and colors  Memorization time – 10  Recall time – 10  Number of figures – 6  Order of recall in the recall stage- random. |
| Under Pressure | Three types of stimuli (a red circle, a black cross, and a letter) will appear one after the other at different spots, anywhere on the screen.  Determine quickly whether the red circle appears above or below the black cross. | Number of stimuli – 20  Time of letter display – 800  Time of cross display – 800  Time of circle display – 200  Time lapse between stimuli – 800  Distance between stimuli – medium, with a disrupting element  . |
